# Prognostic Value of a Ten-Gene Signature in HNSCC Patients Based on Tumor-Associated Macrophages Expression Profiling

**DOI:** 10.3389/fonc.2020.569002

**Published:** 2020-11-18

**Authors:** Zhaoyi Lu, Xiaoli Deng, Hui Li

**Affiliations:** ^1^ Department of Otolaryngology, Head and Neck Surgery, Xiangya Hospital, Central South University, Changsha, China; ^2^ Department of Otolaryngology, The First Affiliated Hospital of Bengbu Medical College, Bengbu, China; ^3^ Textile College, Changzhou Vocational Institute of Textile and Garment, Changzhou, China; ^4^ Key Laboratory of Xinjiang Phytomedicine Resource and Utilization, Ministry of Education, Shihezi University, Shihezi, China

**Keywords:** head and neck squamous cell carcinoma, tumor-associated macrophages, transcription factors, gene signature, prognostic value

## Abstract

Tumor-associated macrophages (TAMs) are regarded as the most abundantly infiltrating immune cells around the tumor microenvironment (TME) in head and neck squamous cell carcinoma (HNSCC), which plays an essential role in immunosuppression and tumorigenesis. In the TCGA HNSCC cohort, 500 patients with clinical-pathological information and RNA sequence expression were randomly assigned to training for lasso regression and validation for verification, respectively. A TAM-based ten-gene signature (TBGs) was constructed, which divided the patients into high-risk and low-risk groups, could predict overall survival (OS) of HNSCC patients in the training dataset (*p* = 3.527e^–05^) and validation dataset (*p* = 3.785e^–02^). The result of Cox univariate and multivariate regression analyses showed that the risk score of TBGs could be an independent prognostic factor in HNSCC. ROC curve confirmed that the risk score of TBGs has good sensitivity and specificity for prognosis prediction (AUC = 0.659) and was also verified by the validation dataset (AUC = 0.621). We obtained key risk transcription factors (TFs)—EHF and SNAI2—by correlation analysis with TBGs. Moreover, we ran a gene set enrichment analysis (GSEA) to speculate that TBGs act on interstitial remodeling, tumor killing, metabolic reprogramming, and tumor immune-related pathways. Finally, we combined clinical–pathological features and risk score of TBGs to establish clinical nomograms, and calibration curves verified the accuracy of long-term clinical prognosis in the two datasets (C-index of 5-year OS = 0.721 and 0.716). In general, the TBGs we obtained may accurately predict the prognosis of HNSCC patients to provide personalized treatment.

## Introduction

The global incidence of head and neck squamous cell carcinoma (HNSCC) continues to rise, which may be related to an increased human papillomavirus (HPV) infection rate, not only due to smoking and drinking (1, 2). Despite the continuous advancement of surgery, radiotherapy, chemotherapy, and immunotherapy, the 5-year survival rate of HNSCC has not substantially improved due to uncontrolled tumor metastasis (3, 4). In recent years, the mechanism of the complex interaction between tumors and immune cells on tumor progression has been continuously explored in the tumor microenvironment (TME) (5). Tumor-associated macrophages (TAMs) account for about 50% of solid tumors and were related to the poor prognosis of tumors, except colon cancer (6). TAMs directly or indirectly affect tumor growth, invasion and metastasis, angiogenesis, immunosuppression, and cancer treatment (7). The heterogeneity of TAMs has always been a difficulty in the study of tumor progression, and TAMs with different phenotypes performed their functions. Scholars have classified macrophages into M1-like type (pro-inflammatory and anti-cancer) and M2-like type (immunosuppressive and pro-cancer) (8). Previous researchers tend to define TAMs as M2-like type (9). In recent years, researchers found that TAMs do not merely express the specific indicators of M1 or M2 type, but more showed the complex of them (10). Nevertheless, scholars still use the M1/2 classification method for the convenience and intuitiveness of research.

Giuseppe et al. (11) reviewed immunohistochemical studies of HNSCC and showed that pan-macrophage indicator CD68 has no prognostic value, and M2 type indicator CD163 predicts a poor prognosis. Ayan et al. (12) found that increased CD68+ and CD163+ density were related to poor clinicopathological indicators and outcome (advanced T stage, nodal metastasis, higher rate of vascular invasion, higher rate of lymphatic invasion, and poor differentiation of tumor). Marker genes of macrophage (MGMs), including infiltration density and polarization-related genes, are significant for the prognosis of HNSCC patients.

In this article, we use bioinformatics to explore the relationship between MGMs and the prognosis of patients in The Cancer Genome Atlas (TCGA) HNSCC database. We found that the lasso regression model based on MGMs can predict the prognosis of HNSCC patients, and the survival time of patients with high-risk coefficients was significantly shorter. Through the verification of the segmentation database, we reckoned the ten-MGMs to be biological prognostic indicators and potential therapeutic targets for HNSCC.

## Materials and Methods

### Data Collection and Analysis

The RNA sequence expression profile data (*n* = 547) and clinical data (*n* = 530) of HNSCC were downloaded from the TCGA database (https://cancergenome.nih.gov/). The transcriptome data included 501 tumor samples, 2 lymph node samples, and 44 paracancerous samples. After the patient ID deduplication, we took the intersection with clinical-pathological data and obtained 500 patients for further analysis. All 500 patients were randomly divided into two datasets *via* R package “caret”: training dataset (*n* = 250) and validation dataset (*n* = 250). The corresponding grouping information and clinical characteristics data are shown in **Supplementary File 1** and **Table 1**.

**Table 1:** HNSCC patients’ clinical characteristics of training dataset and validation dataset in TCGA (*n* = 500).

Characteristic	Training dataset (*n* = 250)	Validation dataset (*n* = 250)	*P* value (chisq. test)
Age (year), *n* (%)			0.719
<60	113 (45.2)	108 (43.2)	
≥60	137 (54.8)	142 (56.8)	
Gender, *n* (%)			0.418
Female	71 (28.4)	62 (24.8)	
Male	179 (71.6)	188 (75.2)	
Histologic grade, *n* (%)			0.146
G1–2	191 (76.4)	172 (68.8)	
G3–4	53 (21.2)	68 (27.2)	
Gx	6 (2.4)	10 (4.0)	
Clinical stage, *n* (%)			0.208
I–II	61 (24.4)	53 (21.2)	
III–IV	185 (74.0)	187 (74.8)	
NA	4 (1.6)	10 (4.0)	
T classification, *n* (%)			0.424
T1–2	94 (37.6)	82 (32.8)	
T3–4	151 (60.4)	158 (63.2)	
Tx	4 (1.6)	7 (2.8)	
NA	1 (0.4)	3 (1.2)	
N classification, *n* (%)			0.797
N0	119 (47.6)	119 (47.6)	
N1–3	121 (48.4)	119 (47.6)	
Nx	9 (3.6)	9 (3.6)	
NA	1 (0.4)	3 (1.2)	
M classification, *n* (%)			0.308
M0	235 (94.0)	235 (94.0)	
M1	4 (1.6)	1 (0.4)	
Mx	10 (4.0)	10 (4.0)	
NA	1 (0.4)	4 (1.6)	
Vital status, *n* (%)			0.290
Alive	141 (56.4)	141 (56.4)	
Dead	109 (43.6)	109 (43.6)	

HNSCC, head and neck squamous cell carcinoma; TCGA, The Cancer Genome Atlas.

### Sources of Macrophage-Related Gene and TF Data

We extracted 54 M1-like type genes and 43 M2-like type genes from differential expression genes of macrophage polarization by Fernando et al. (13). From research on the immune system of human cancer, 33 infiltration genes of TAMs were acquired (14). We downloaded TF data from the Cistrome Project (http://cistrome.org/). The 130 MGMs and 318 TFs are listed in **Supplementary File 2**.

### Construction of Prognostic Signature for TCGA HNSCC Data and Prognostic Value of Risk Score

All statistical analysis and model building are based on software R (version 3.6.0), and all differentially expressed genes (DEGs) with |log2 fold change (FC)| ≥ 1 and adjusted *P* values <0.05 after judgment by R package “limma” were selected for subsequent analysis. The visual heatmaps and the volcano maps of DGEs were done using the R package “pheatmap” and “ggplot2.” Based on univariate Cox regression, we established the best lasso model using the screened MGMs (15). The risk score was calculated as follows: $\text{risk score }=\sum_{j=1}^{n}\text{Coefj }*\text{Xj}$. *Coefj* is the coefficient and *Xj* is the relative gene expression *via* z-score standardized reckon. The R function “cor.test” was used to calculate and test the correlation coefficient in which the filter conditions were cor >0.4 and pvalue <0.001. According to the HR value calculated by R function “coxph,” we assigned MGMs into two groups: HR > 1 were identified as risk genes and HR < 1 were protective genes. The regulation network of differentially expressed TFs on the risk or protective MGMs was visualized by R package “ggalluvial.” Kaplan–Meier and log-rank methods were used to analyze whether the risk score was related to the prognosis of HNSCC patients. Univariate and multivariate Cox tests were performed to investigate the correlation between risk curves and OS. The receiver operating characteristic (ROC) curve was drawn by the R package “survivalROC” to evaluate the sensitivity and specificity of clinical parameters and the risk score for prognosis prediction by the area under curve (AUC) value.

### A Clinical Prognostic Prediction Model

As an excellent predictive model for tumor prognosis (16), a nomogram can provide intuitive help for clinical prediction. Based on clinical parameters (age, gender, grade, TNM stage, T, N, and M classification) and risk score, we plotted the nomogram to predict the probability of 1-, 3- and 5-year OS with the R package “rms.” The accuracy of the 5-year survival rate prediction is verified by calculating the concordance index (C-index) of the training dataset and the validation dataset. Value 0.5 and 1.0 of the C-index respectively predict the random and excellent accuracy of the nomogram.

### Gene Set Enrichment Analysis

We used software GSEA 4.0.3 to generate statistical differences between two biological expression states according to the high- and low-risk score. Results reveal the critical signaling pathways and biological processes involved, providing the research direction for the next mechanism experiment (17). Each gene set was calculated 1000 times and then screened by their nominal *p*-value and normalized enrichment score (NES).

## Results

### Difference Analysis of Training Dataset

**Figure 1:**
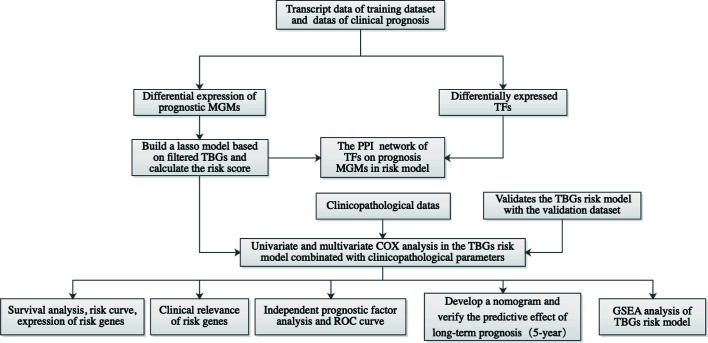
Workflow graph for this study. MGMs, marker genes of macrophage; TBGs, TAM-based ten-gene signatures; TFs, transcription factors; PPI, protein–protein interaction; ROC, receiver operating characteristic; GSEA, gene set enrichment analysis.

**Figure 2:**
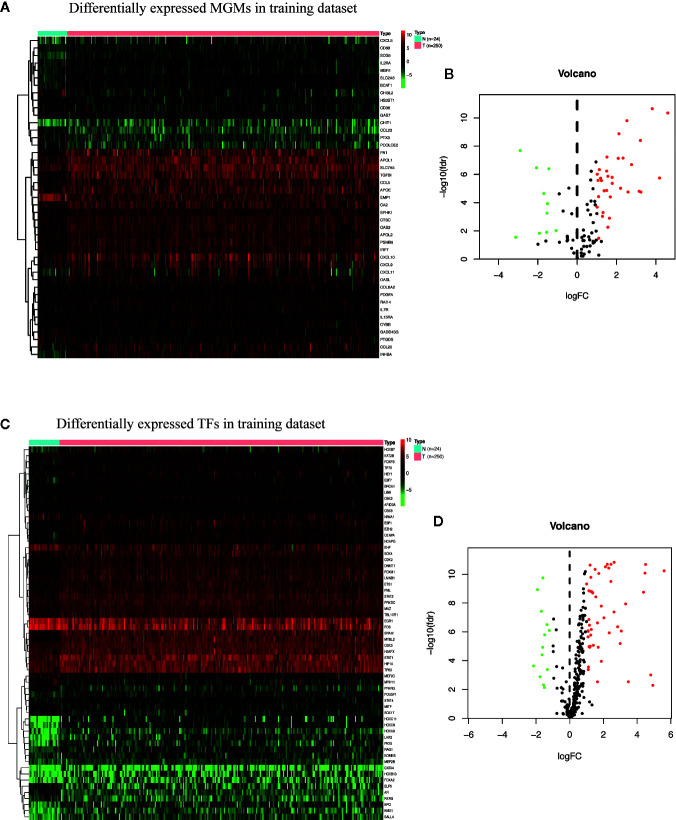
Identification of differentially expressed MGMs and TFs in the training dataset. **(A, B)** Heat map and volcanic map of 43 differentially expressed MGMs in the training dataset. **(C, D)** Heatmap and volcanic map of 61 differentially expressed TFs in the training dataset.

The flow chart of this research is shown in **Figure 1**. First, we got all differentially expressed genes *via* the difference analysis of 250 tumor tissues and 24 tumor-adjacent tissues in the training dataset. Then we extracted the differential gene expression profiles of 43 MGMs and 61 TFs (**Supplementary File 3**) to draw the heatmaps and volcano plots (**Figures 2A–D**).

### Prognosis-Related Macrophage Genes

**Figure 3:**
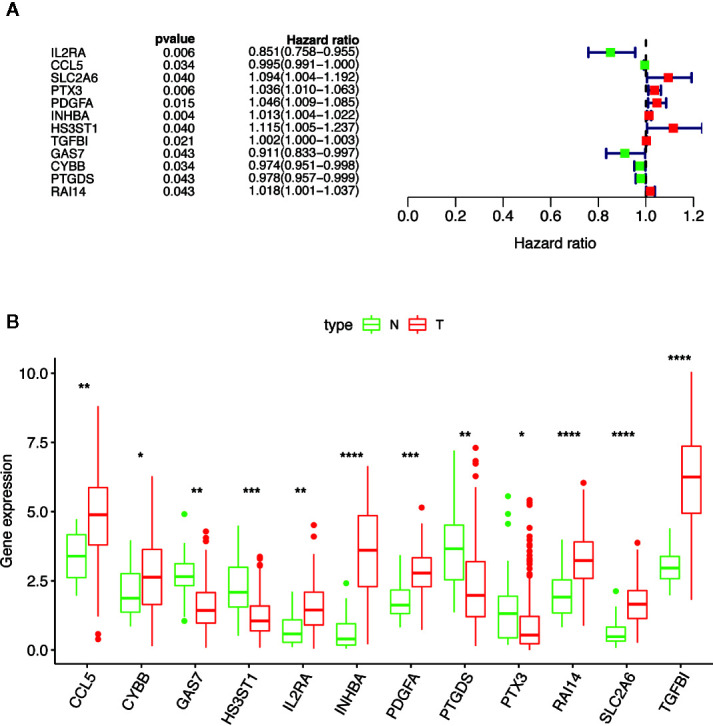
Selection of 12 MGMs associated with the OS of HNSCC patients by univariate Cox regression analysis. **(A)** Univariate Cox regression analysis. Forest plot of 12 MGMs associated with survival. **(B)** Differential expression of the 12 MGMs between 24 tumor-adjacent tissues and 250 HNSCC tissues. Note: *P < 0.05, **P < 0.01, ***P < 0.001, ****P < 0.0001.

Combined with the clinical information of the patients, Cox univariate regression analysis was used to screen out the prognostic MGMs associated with the OS of TCGA-HNSC (**Figure 3A**) and determine their expression level in the training dataset (**Figure 3B**).

### Construction of Prognosis-Related MGMs Lasso Regression Model

**Figure 4:**
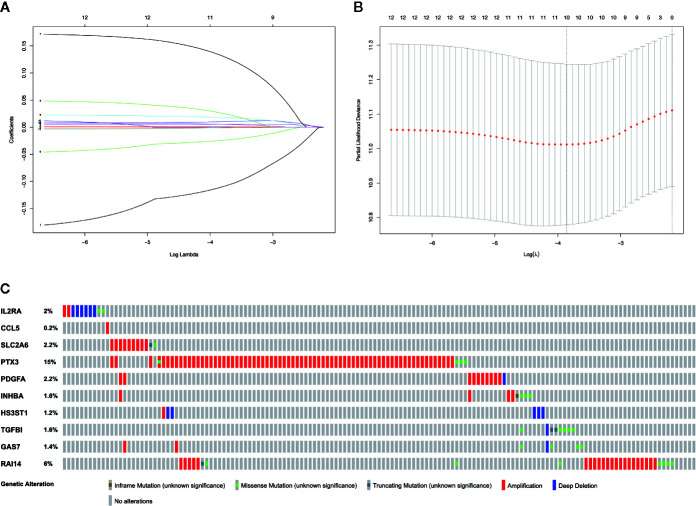
Establishment of prognostic gene signature by LASSO regression analysis. **(A)** LASSO coefficient profiles of the 12 prognostic MGMs in the training dataset. **(B)** A coefficient profile plot was generated against the log (lambda) sequence. **(C)** Genetic alteration of the ten genes in the TCGA-HNSCC cohort (TCGA, Firehose Legacy).

Based on the aforementioned twelve prognostic MGMs, we ran the lasso regression model and calculated the regression coefficient. The coefficient of MGMs is shown in **Figure 4A**. The model can achieve the best fit when 10 of 12 MGMs are included (**Figure 4B**). The function of the ten MGMs, including the role of TAMs in regulating the TME through metabolism, inflammation, and immunosuppression, the risk coefficient of which is shown in **Table 2**. At the same time, we found the ten MGMs were altered in 142 (28%) of the queried patients in the database (HNSC, Firehose Legacy, http://www.cbioportal.org), indicating that the ten MGMs play a vital role in the progress of HNSCC (**Figure 4C**).

**Table 2:** Functions of MGMs in the prognostic gene signatures.

No.	Gene symbol	Full name	Function	Risk coefficient
1	IL2RA	The interleukin 2 (IL2) receptor alpha	Result from extracellular proteolysis	–0.0970655
2	CCL5	Chemokine ligand 5	Involved in immunoregulatory and inflammatory processes	–0.0008015
3	SLC2A6	Solute carrier family 2 member 6	Hexose transport	0.01694515
4	PTX3	Pentraxin 3	Response to inflammatory stimuli	0.01096275
5	PDGFA	Platelet-derived growth factor alpha polypeptide	Regulates developmental processes and alternative splicing	0.01629422
6	INHBA	Inhibin beta A subunit	Associated with cancer cachexia	0.00528319
7	HS3ST1	Heparan sulfate 3-O-sulfotransferase 1	Biosynthetic enzymes with biologic activities	0.11410459
8	TGFB1	Transforming growth factor beta-induced 68kDa	Inhibit cell adhesion	0.00067328
9	GAS7	Growth arrest specific 7	Growth arrest	–0.0204874
10	RAI14	Retinoic acid-induced protein 14	Development of different tumor types	0.00429914

### Risk Prognostic Networks of Differentially Expressed TFs and Differentially Prognostic MGMs

**Figure 5:**
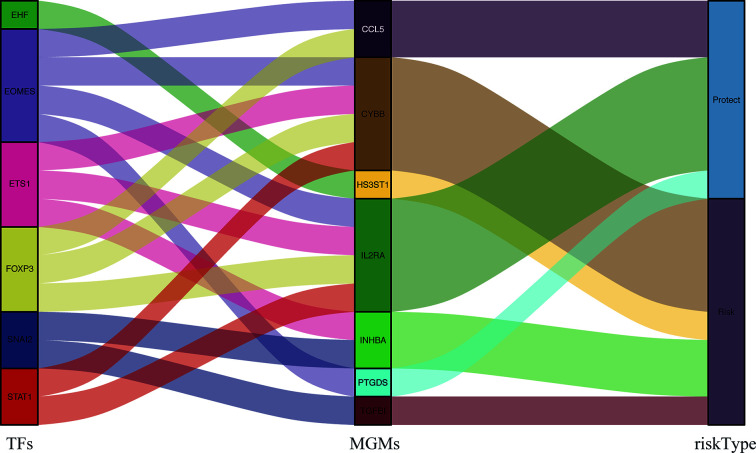
PPI between differentially expressed TFs and the prognostic MGMs with protective or risk type in the Sankey diagram.

The correlation between differentially expressed TFs and prognostic MGMs can be seen in **Supplementary File 4**, in which genes (CYBB, HS3ST1, INHBA, TGFB1) with HR > 1 are considered to be risk genes, and genes (CCL5, IL2RA, PTGDS) with HR < 1 were protective genes. We used the Sankey diagram to depict the regulatory relationship between TFs on risk and protective MGMs (**Figure 5**). Among them, EHF regulates risk gene HS3ST1 and SNAI2 regulates risk genes INHBA and TGFB1, while EOMES, ETS1, FOXP3, and STAT1 regulate both risk genes and protective genes.

### Survival Analysis, Risk Curve, and Risk Gene Profiles of HNSCC Patients

**Figure 6:**
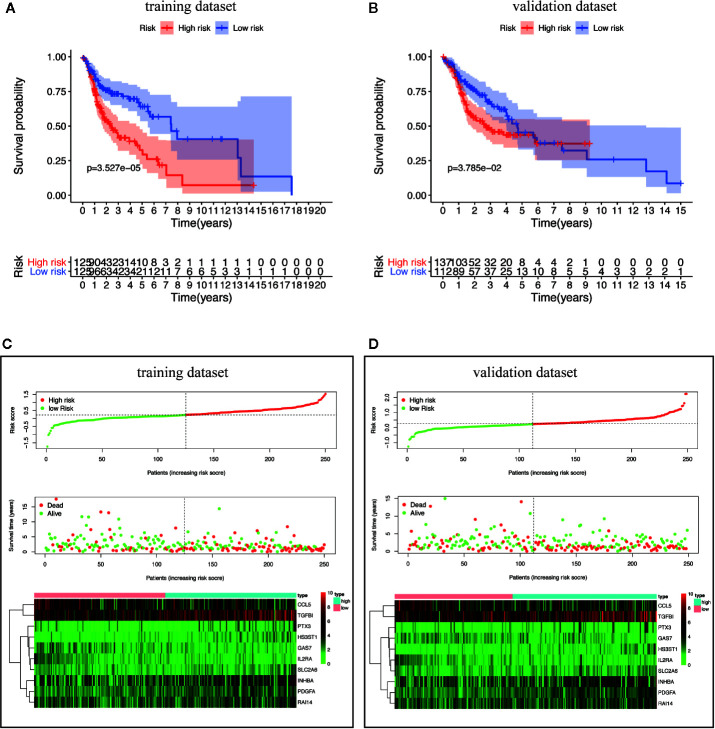
Survival analysis and characteristics of the TBGs. **(A, B)** Kaplan–Meier survival curves of overall survival between the high-risk group and low-risk group in the training dataset and validation dataset. **(C, D)** The distribution of risk score, patient’s survival time and status, as well as TBGs expression profiles for the training dataset and validation dataset.

By scoring each patient’s risk through the model and dividing the training dataset into a high-risk group (*n* = 125) and a low-risk group (*n* = 125), we found that the survival time of the high-risk group was significantly lower (*p* = 3.527e^−05^) (**Figure 6A**). The risk score, survival status distribution of the patients and the expression of risk genes in the training data group are shown in **Figure 6B**. We got consistent results (*p* = 3.785e^–02^) from the validation dataset that verify the model consistency (**Figures 6C, D**).

### Clinical Relevance and Independent Prognosis Analysis

**Figure 7:**
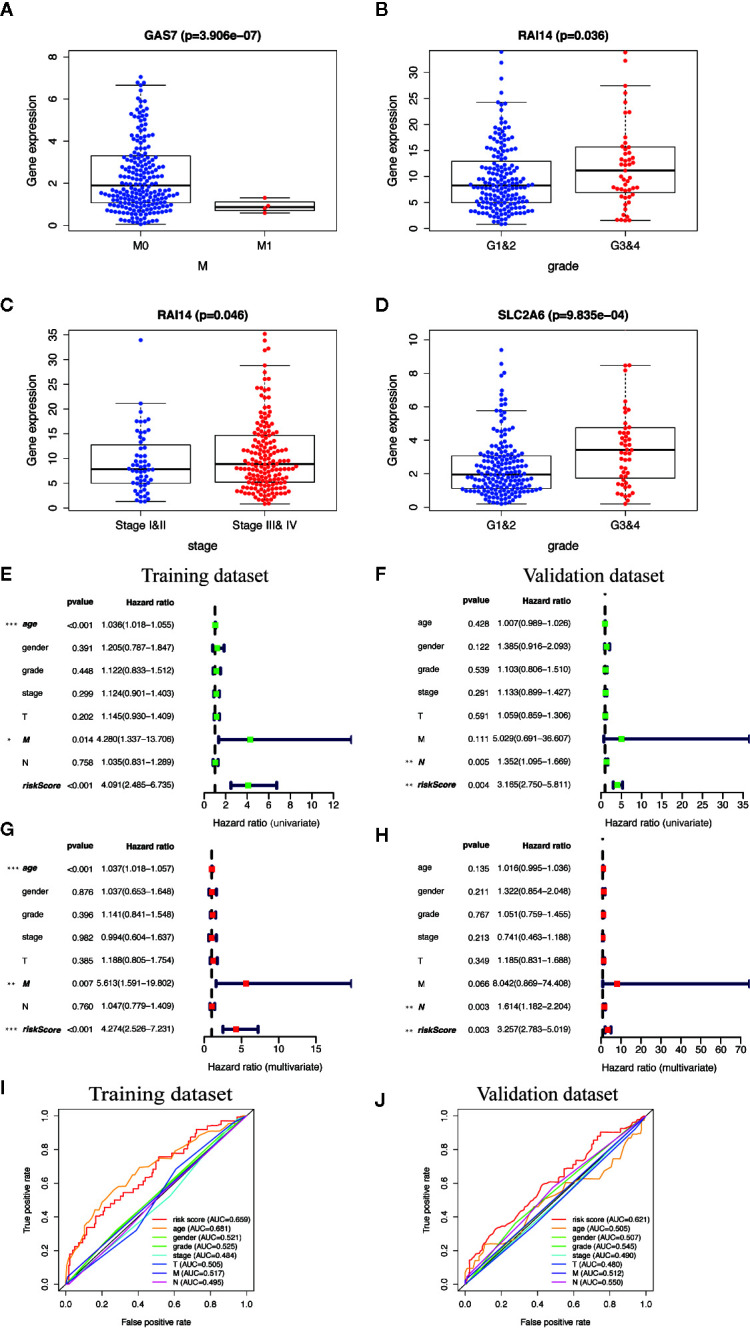
Clinical correlation analysis and independent prognosis analysis of TBGs and clinicopathologic characteristics. **(A)** Differentially expressed GAS7 in M classification (M0 and M1). **(B, C)** Differentially expressed RAI14 in pathologic grade classification (G1–2 and G3–4) and TNM classification (Stage I–II and Stage III–IV). **(D)** Differentially expressed SLC2A6 in pathologic grade classification (G1–2 and G3–4). **(E, F)** Univariate Cox regression analysis. Forest plot of the association between risk factors and survival of training dataset and validation dataset. **(G, H)** Multivariate Cox regression analysis. Forest plot of the association between risk factors and survival of training dataset and validation dataset. **(I, J)** Receiver operating characteristic (ROC) analysis of the sensitivity and specificity of the OS for the ten-gene risk score in the training dataset and validation dataset. Note: *P < 0.05, **P < 0.01, ***P < 0.001, ****P < 0.0001.

We analyzed the correlation between the ten MGMs and clinicopathological characteristics (age, gender, grade, TNM stage, T, N, and M classification). Results showed that GAS7 expression was associated with inhibition of tumor metastasis (*p* = 3.906e^−07^), RAI14 expression was associated with high pathological grade (*p* = 0.036) and advanced clinical stage (*p* = 0.046), and SLC2A6 expression was associated with high pathological grade (*p* = 9.835e^−04^) (**Figures 7A–D**). Cox univariate and multivariate regression analysis showed that the training dataset and validation dataset have different clinical prognostic indicators, but the risk score can be used as potential independent prognostic indicators in two datasets (*P* < 0.05) (**Figures 7E–H**). The ROC curve is drawn to show that the sensitivity and specificity of the risk score also have a relatively good performance in prognostic prediction (AUC = 0.659 in training dataset) and (AUC = 0.621 in validation dataset) (**Figures 7I, J**). Overall, these results confirmed that this TBGs based on the ten MGMs was also predictive of survival in the independent validation HNSCC cohorts.

### Construction of a Clinical Prognostic Prediction Model

**Figure 8:**
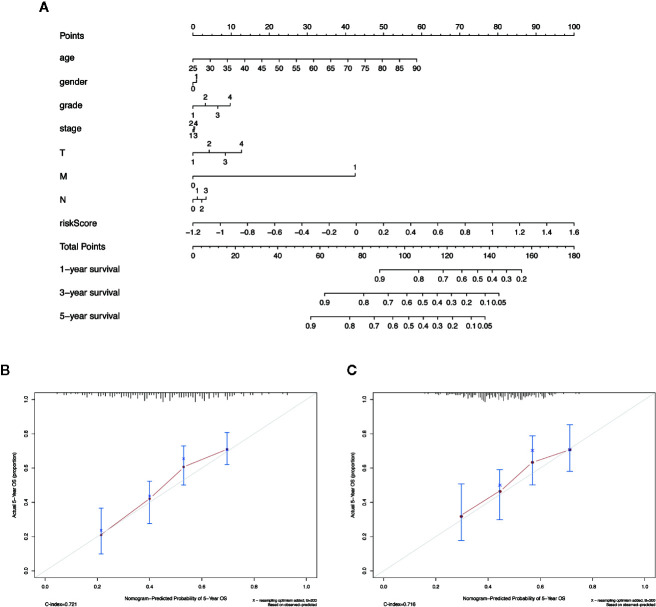
The nomogram to anticipate prognostic probabilities in HNSCC dataset. **(A)** The nomogram for predicting OS development in the training dataset. **(B, C)** The calibration plots for predicting 5-year in the training dataset and validation dataset. Notes: The x-axis and y-axis represent nomogram predicted and actual survival, respectively. The solid line indicates the predicted nomogram, and the vertical bars represent a 95% confidence interval.

We integrated the risk score and established a nomogram to facilitate our risk model application in clinical prognosis (**Figure 8A**). By calculating the score of each feature of patients, we can predict the 1-, 3-, and 5-year OS probability, contributing to precision treatment. In the calibration curves of the training dataset and the validation dataset, the C-index of the 5-year OS rate prediction is 0.721 and 0.716, respectively (**Figures 8B, C**). We hold the opinion that the nomogram may have good accuracy for long-term survival prediction in HNSCC.

### GSEA Identifies MGMs-Related Signaling Pathway

**Figure 9:**
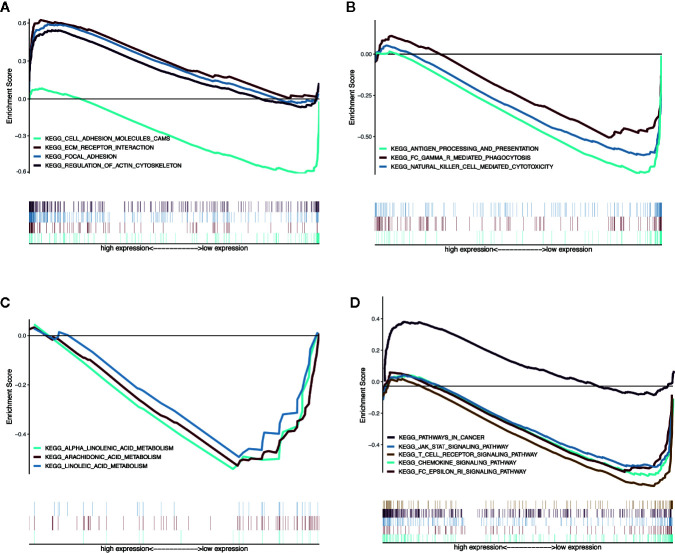
Enrichment plots from multiple GSEA. **(A)** Intercellular matrix remodeling: cell adhesion molecules cams, focal adhesion, ECM receptor interaction, regulation of actin cytoskeleton. **(B)** Tumor killing: antigen processing and presentation, Fc gamma r mediated phagocytosis, natural killer cell mediated cytotoxicity. **(C)** Metabolic reprogramming: alpha linoleic acid metabolism, arachidonic acid metabolism, linoleic acid metabolism. **(D)** Tumor immune-related pathways: chemokine signaling pathway, Fc epsilon RI signaling pathway, JAK-STAT signaling pathway, T-cell receptor signaling pathway, pathways in cancer.

We used GSEA to analyze the differences in the pathways involved in the groups with high- and low-risk scores to understand the potential mechanism. We screened out enrichment results that meet the criteria (FDR < 0.25, NOM *p*-value < 0.05) in the MSigDB gene set (c2.cp.kegg.v6.2.symbols.gmt) (**Table 3**). Based on the NES value, significantly enriched pathways are filtrated, mainly in four fields related to TAM function: intercellular matrix remodeling, tumor killing, metabolic reprogramming, and tumor immune-related pathways (**Figures 9A–D**).

**Table 3:** Gene sets enriched in risk model.

MSigDB collection	Gene set name	NES	NOM p-val	FDR q-val
c2.cp.kegg. v6.2. symbols.gmt	KEGG_CELL_ADHESION_MOLECULES_ CAMS	–1.857	0.010	0.024
	KEGG_ECM_RECEPTOR_INTERACTION	1.708	0.019	0.235
	KEGG_FOCAL_ADHESION	1.791	0.014	0.219
	KEGG_REGULATION_OF_ACTIN_CYTOSKELETON	1.566	0.033	0.244
	KEGG_ANTIGEN_PROCESSING_AND_PRESENTATION	–2.131	0	0.006
	KEGG_FC_GAMMA_R_MEDIATED_PHAGOCYTOSIS	–1.55	0.035	0.203
	KEGG_NATURAL_KILLER_CELL_MEDIATED_CYTOTOXICITY	–1.902	0.011	0.032
	KEGG_ALPHA_LINOLENIC_ACID_METABOLISM	–1.586	0.022	0.174
	KEGG_ARACHIDONIC_ACID_METABOLISM	–1.879	0	0.030
	KEGG_LINOLEIC_ACID_METABOLISM	–1.553	0.039	0.194
	KEGG_CHEMOKINE_SIGNALING_PATHWAY	–1.716	0.018	0.086
	KEGG_FC_EPSILON_RI_SIGNALING_PATHWAY	–1.778	0.002	0.054
	KEGG_JAK_STAT_SIGNALING_PATHWAY	–1.519	0.042	0.218
	KEGG_PATHWAYS_IN_CANCER	1.944	0.037	0.247
	KEGG_T_CELL_RECEPTOR_SIGNALING_PATHWAY	–1.823	0.014	0.040

Gene sets with NOM P-value <0.05 and FDR q-value <0.25 are considered as significant.

FDR, false discovery rate; NES, normalized enrichment score; NOM, nominal.

## Discussion

Recent studies have found that TAMs cannot be categorized only as M1-like or M2-like type, but as their complexes, so it is more effective to integrate macrophage characteristics as prognostic factors. Specific subtypes of TAMs in the TME of HNSCC have their corresponding functions, and their respective divisions facilitate tumors with phenotypic subsets of EMT, stemness, and immunosuppression through cell–cell communication. In the protein–protein interaction (PPI) between differentially expressed TFs and the prognostic MGMs, we get the risk genes and the protective genes to maintain the accuracy of prognosis prediction to provide precise treatment. Among the risk genes, HS3ST1 and TGFB1 belong to M2-type marker genes, INHBA is a M1-type marker gene, and CYBB is an infiltration density gene. CCL5 and IL2RA are M1 marker genes; PTGDS is an infiltration density gene in the protective genes.

Heparin sulfate (HS) proteoglycans are a vital part of the cell microenvironment. The fine structure of the polysaccharide HS chain plays an essential role in cell–cell interaction, adhesion, migration, and signal transduction. Previous studies have confirmed that the HS biosynthesis system is intimately involved in the carcinogenesis process. The glycosylated protein HS3ST1 in ER-negative breast cancers contributes to tumor metastasis (18) and is also a biological prognostic indicator of glioma (19), prostate cancer (20), and acute lymphocytic leukemia. The immunosuppressive gene TGFB1 is highly expressed in HNSCC, and the expression level is higher in lymph nodes (21). In HPV^+^ HNSCC, TGFB1 is expressed higher in HPV 33^+^ compared with HPV16^+^ patients with less CD8^+^ T cell infiltration and worse prognosis (22). In HNSCC patients who received chemoradiotherapy, regardless of the severity of complications (mucosal inflammation), the single nucleotide polymorphism (SNP) rs1982073 of the TGFB1 gene is associated with prognosis (23). The higher expression of inhibin subunit beta A (INHBA) is related to the poor disease-free survival of HNSCC patients (24). Mechanism exploration found that INHBA can accelerate the lymph node metastasis by regulating the RUNX2 signaling pathway (25). CYBB is a primary component of the microbicidal oxidase system of phagocytes and associated with smoking risk factors in patients with lung adenocarcinoma (26), which is involved in encoding NOX2 to promote lung cancer metastasis (27). Prostaglandin D2 (PGD2) synthase (PTGDS) and its receptor PTGDR2 are negatively correlated with stem genes (Sall4 and Lgr5) in gastric cancer, which restricts tumor self-renewal, growth, and metastasis by relying on the PTGDR2 pathway to inhibit STAT3 phosphorylation and nuclear expression (28). PGD2 in intestinal tumors also mediated the anti-cancer effect through its receptor (29). Inflammatory factors produced by endothelial cells promote PTGDS expression and release PGD2, which inhibited malignant biological behavior of vascular permeability, angiogenesis, EMT, and tumor apoptosis (30). CCL5, a specific chemokine released by macrophages, regulates inflammation, of which its role in tumor progression is controversial (31). Knockdown of glycogen branching enzyme (GBE1) downstream of HIF1 in lung adenocarcinoma led to an increase in CCL5 expression and recruited more cytotoxic CD8^+^ T lymphocytes to contribute to tumor regression (32). The IL2Ra level in serum may be meaningful for prognosis after cancer treatment, which increased by 63.8% in HNSCC patients (*p* = 0.032) after cisplatin chemoradiation in Panagiota’s report (33).

The heterogeneity of TFs in different TMEs is enormous and of great significance (34). Our correlation analysis revealed some key TFs that regulate MGMs. We found that specific TFs can regulate both risk genes and protective genes, which is consistent with the conclusion in recent years that *in vivo* experiments found that the activity of TAMs is regulated by multiple transcription processes (35). However, EHF and SNAI2 only regulate the expression of risk genes, which deserves attention. TBGs may also be related to the pathological grade, TNM stage, and metastasis of HNSCC, and may play a role in different carcinogenic mechanisms. Results of GSEA suggested that MGMs regulate intercellular substance remodeling, tumor killing, metabolic reprogramming, and tumor immune-related pathways to affect the progression of HNSCC.

## Conclusion

In summary, we found that a gene signature contains the ten macrophage-related genes in HNSCC and analyzed their possible independent prognostic value. Also, we built a risk model based on the ten MGMs and a nomogram that can be used to predict long-term survival in the clinic. The downside is that we did not find a dataset in the GEO database for HNSCC that contains complete RNA sequence information of TBGs, which might lead to a lack of confidence in our model. Therefore, more prospective studies need to be included in the future to verify the predictive ability of this feature in clinical applications.

## Data Availability Statement

Publicly available datasets were used in this study. The RNA sequence and clinical data of HNSCC were downloaded from the TCGA database (https://cancergenome.nih.gov/).

## Author Contributions

HL was responsible for project design. ZL analyzed data and wrote the manuscript, and XD participated in manuscript editing and polishing. All authors contributed to the article and approved the submitted version.

## Funding

This work was funded by the Anhui Provincial Key Research and Development Project (202004j07020007), Anhui Provincial Natural Science Foundation (1608085MH236), Key Project of Natural Science of Bengbu Medical College (BYKY2019086ZD) and Science Research Project of Bengbu Medical College (BYKF1855).

## Conflict of Interest

The authors declare that the research was conducted in the absence of any commercial or financial relationships that could be construed as a potential conflict of interest.

## Supplementary Material

Supplementary File 1 Grouping information of training dataset and validation dataset.

Supplementary File 2 List of MGMs and TFs in this research.

Supplementary File 3 The differential gene expression profiles of 43 MGMs and 61 TFs.

Supplementary File 4 Correlation analysis of differential TFs on the prognosis MGMs.
